# Higher intensity walking improves global cognition during inpatient rehabilitation: a secondary analysis of a randomized control trial

**DOI:** 10.3389/fneur.2023.1023488

**Published:** 2023-06-09

**Authors:** Sue Peters, Keith R. Lohse, Tara D. Klassen, Teresa Liu-Ambrose, Sean P. Dukelow, Mark T. Bayley, Michael D. Hill, Sepideh Pooyania, Jennifer Yao, Janice J. Eng

**Affiliations:** ^1^School of Physical Therapy, University of Western Ontario, London, ON, Canada; ^2^Program in Physical Therapy and Department of Neurology, Washington University School of Medicine, Saint Louis, MO, United States; ^3^Department of Physical Therapy, University of British Columbia, Vancouver, BC, Canada; ^4^Centre for Aging SMART at Vancouver Coastal Health, Vancouver, BC, Canada; ^5^Department of Clinical Neurosciences and Hotchkiss Brain Institute, University of Calgary, Calgary, AB, Canada; ^6^Division of Physical Medicine and Rehabilitation, University of Toronto, Toronto, ON, Canada; ^7^Division of Physical Medicine and Rehabilitation, University of Manitoba, Winnipeg, MB, Canada; ^8^Division of Physical Medicine and Rehabilitation, University of British Columbia, Vancouver, BC, Canada

**Keywords:** rehabilitation, cognition, gait, outcomes, stroke, exercise

## Abstract

Cognitive deficits are common poststroke. Cognitive rehabilitation is typically used to improve cognitive deficits. It is unknown whether higher doses of exercise to promote motor recovery influence cognitive outcomes. Our recent trial, Determining Optimal Post-Stroke Exercise (DOSE), shows more than double the steps and aerobic minutes can be achieved during inpatient rehabilitation versus usual care, and translates to improved long-term walking outcomes. Thus, the secondary analysis aim was to determine the effect of the DOSE protocol on cognitive outcomes over 1-year poststroke. The DOSE protocol progressively increased step number and aerobic minutes during inpatient stroke rehabilitation over 20 sessions. The Montreal Cognitive Assessment (MoCA), Digit Symbol Substitution Test (DSST), and Trail Making Test B were completed at baseline, post-intervention, and 6- and 12-months poststroke, administered using standardized guidelines. Using the DOSE data, we used mixed-effect spline regression to model participants’ trajectories of cognitive recovery, controlling for relevant covariates. Participants (Usual Care *n* = 25, DOSE *n* = 50) were 56.7(11.7) years old, and 27(10) days post stroke. For the MoCA, there were statistically significant Group × Trajectory(*p* = 0.019), and Group × ΔTrajectory (*p* = 0.018) interactions with a substantial clinically meaningful difference, from +5.44 points/month improvement of the DOSE group compared to +1.59 points/month improvement with Usual Care during the 4-week intervention. The DSST and Trails B improved over time but were not different between groups. Taking advantage of this early difference may lend support to continued efforts to increase intensity, during and after discharge from inpatient rehabilitation, to improve cognition.

**Clinical trial registration**: www.clinicaltrials.gov, NCT01915368.

## Introduction

Though motor recovery is a primary focus within the early stage of stroke rehabilitation, cognitive deficits are common (1). Cognitive rehabilitation is typically used to improve cognitive deficits (2). It is unknown whether higher doses of exercise to promote motor recovery have an effect on cognitive outcomes. There are several mechanisms through which exercise may improve brain and cognitive function, such as increases to hippocampal volume (3), greater levels in the serum of brain-derived neurotrophic factor (3), and increased gray and white matter volume in temporal and prefrontal brain regions (4). Our recent study shows more than double the steps and aerobic minutes can be achieved during inpatient rehabilitation versus usual care, and translates to improved long-term walking outcomes (5). Thus, the aim of this secondary analysis was to determine the effect of this protocol on cognitive outcomes over 1-year poststroke.

## Methods

These data are from the Determining Optimal Post-Stroke Exercise (DOSE) trial [2014–2018 (5)]. The DOSE protocol increased step number and aerobic minutes with walking-related, weight-bearing activities, with clinical improvements in walking outcomes over usual care (5). The protocol completed ≥30 min of activities that progressed in step number and aerobic minutes over 20 sessions. Protocol details are at https://neurorehab.med.ubc.ca/, and in the primary paper (5). The clinical trial consisted of Usual Care (Group 1) with five 1-h sessions per week = 20 h, DOSE1 (Group 2) intervention replaced physical therapy for five 1-h sessions per week for 20 sessions (total 20 h but double step number and aerobic minutes), and DOSE2 (Group 3) received an extra, 1-h exercise session, 5 days/week, for 4 weeks (totaling 40 h). The primary outcomes paper demonstrated similar walking improvement 1-year poststroke for DOSE1 and DOSE2 (5) so those groups were pooled for this analysis (DOSE group). All participants were cognitively able to provide informed consent. The sample size for this study was determined based on the primary outcomes paper, and therefore the analyses here should be treated as exploratory.

Cognitive outcome measures were completed at baseline (average of 4 weeks post-stroke), post-intervention, and at 6- and 12-months poststroke, administered using standardized guidelines. The Montreal Cognitive Assessment (MoCA) is a measure of global cognitive function, including visuospatial abilities, executive functions, attention, memory, language, and orientation to time and space (/30) (6). While the MoCA is primarily used as a screening tool, it also has acceptable responsiveness to detect changes over time with a minimal clinically importance difference (MCID) of 1.22 (anchor-based) and 2.15 (distribution-based) (1), and hence is valid as an outcome measure for clinical trials. Stroke survivors with score increases above these values may demonstrate clinically important improvements to cognition (1).

The Digit Symbol Substitution Test (DSST) measured information processing speed (higher number indicates better speed). The Trail Making Test B measured set-shifting, and a rate-of-generation score was calculated (total completed responses/total time to complete responses in seconds).

### Statistical analysis

**Figure 1:**
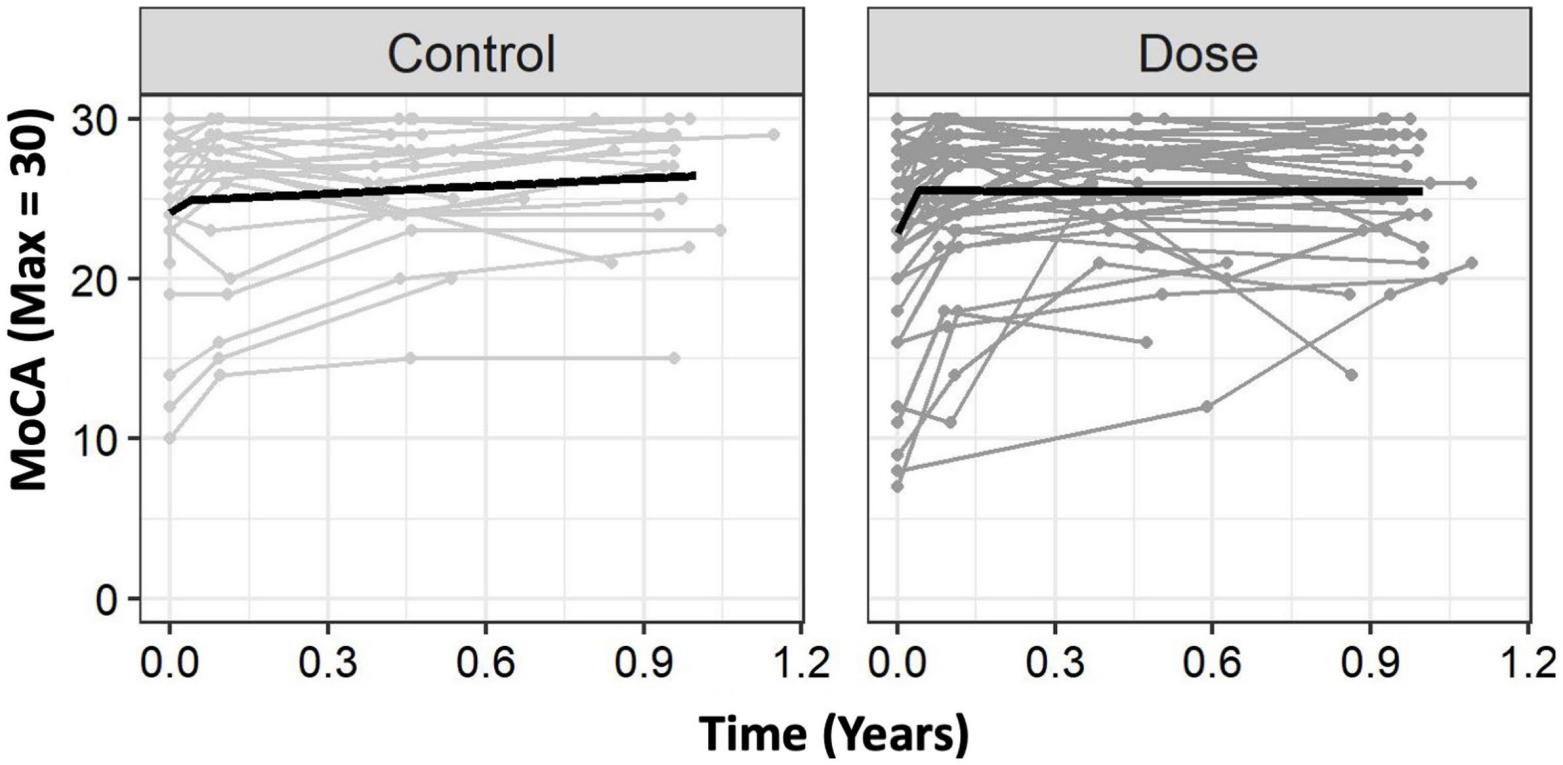
Group level trajectories. The Usual Care group had an initial trajectory (rate of change) of 19.06 points on the MoCA per year. After the knot, this rate of change slowed down to 1.59 points/year. DOSE had a much greater rate of change of 65.38 points/year during the intervention and had minimal change following the knot, slowing to −0.06 points/year. For clinical interpretability, we provide these values in months. For the MoCA, DOSE showed a greater rate of change (+5.44 points/month) compared to Usual Care (+1.59 points/month) prior to the knot (0.04 years), but the DOSE trajectory flattened out more after the knot than it did for Usual Care. Time 0 is time of study enrollment. MoCA, Montreal Cognitive Assessment.

We used mixed-effect regression models to capture change over time (7) which preserved the exact date when outcomes were measured, and accommodated missing observations (Supplementary material). A series of unconditional mixed-effect regression models were fit for each dependent variable to determine the best way to capture change over time (Supplementary material). For all outcomes, we tested a series of linear, quadratic, and spline models with random effects on the intercept and the linear slope of the time variable. Visual inspection of the data suggested strong nonlinearities in cognitive outcomes over time. Specifically, there appeared to be a greater rate of change during the intervention, which tended to plateau following the intervention (Figure 1). To address this nonlinearity, we fit (single-knot) spline models (which can provide a better fit to the data than polynomial models when plateaus are present) (8). Advantages of the mixed-effect regression model include estimating a unique trajectory for each participant (versus focusing on mean differences), preserving variability in time (as no participant is measured at precisely the same time), and allowing change to be expressed as clinically meaningful points/year at different times post-stroke. The Supplementary material provides more detail regarding these methods. Across all dependent variables, a single knot spline model provided the best model fit with the best fitting knot consistently being placed at 0.04 years (14.6 days) across models, which is approximately the midpoint of the 4-week intervention (Supplementary material).

Next, to test the effects of DOSE on cognitive outcomes, we added fixed-effects of Group (Usual Care versus DOSE), and Group × Trajectory (trajectories before the knot) and Group × ΔTrajectory (trajectories changes post knot) interactions to the model. The Trajectory represents the progression or shape of the variable over time. All models controlled for the fixed-effects of sex, age, years of education, and time since stroke as covariates. Statistical significance of regression coefficients was based on deviance change, using the Welch-Satterthwaite approximation of degrees of freedom, with α = 0.05 for all tests (9). To ensure robustness of the results to model assumptions violations, we calculated semiparametric bootstrapped 95% confidence intervals (using *n* = 1,000 simulations) (10).

## Results

Participants (Usual Care *n* = 25, DOSE *n* = 50) were 56.7(11.7) years old, 27(10) days post stroke at randomization; 30 female/45 male; 43 right/32 left hemisphere stroke; and 62 ischemic/13 hemorrhagic, shown as mean (SD) or counts. Seven participants had mild expressive aphasia (Usual Care *n* = 2, DOSE *n* = 5). Table 1 provides baseline data for both groups.

**Table 1:** Baseline data.

Variable	Usual Care (*n* = 25)	DOSE (*n* = 50)
Male (%)	15 (60%)	30 (60%)
Age (y) – Mean(SD)	56.6 (13.7)	56.8 (10.7)
Time since stroke (d) – Mean (SD)	25.5 (10.8)	27.7 (10.2)
Education (y) – Mean (SD)	13.3 (2.67)	14.4 (3.43)
Stroke location – Hemisphere (# right/left)	17/8	26/24
Type of stroke (# ischemic/hemorrhagic)	21/4	41/9
MoCA baseline – Med [IQR], *n*	26 [23, 28], *n* = 25	25 [22, 27], *n* = 50
MoCA post-intervention – Med [IQR], *n*	27 [24, 29], *n* = 25	28 [24, 28], *n* = 49
MoCA 6-months – Med [IQR], *n*	26 [24, 28], *n* = 24	27 [24, 28], *n* = 43
MoCA 12-months – Med [IQR], *n*	28 [25, 29], *n* = 18	26 [24, 29], *n* = 37

MoCA, Montreal Cognitive Assessment; y, years; SD, standard deviation; d, days; IQR, interquartile range.

### MoCA

For the MoCA, there were statistically significant effects of Sex, *F*(171.17) = 4.42,*p* = 0.039, Education, *F*(170.97) = 20.0, *p <* 0.001, and Time Since Stroke (TSS), *F*(170.5) = 8.78, *p* = 0.004, such that females tended to have lower baseline scores than males, individuals with more years of education tended to have higher baseline scores, and individuals who were randomized later following stroke tended to have worse baseline scores. There was not a statistically significant effect of Age, *F*(170.6) = 0.06, *p* = 0.809. There was an effect of Trajectory, *F*(1182.9) = 18.68, *p* < 0.001, and Δtrajectory, *F*(1182.9) = 16.9, *p* < 0.001. There was no significant main-effect of Group, *F*(1105.8) = 1.48, *p* = 0.225, suggesting that groups were similar at enrollment. However, there were statistically significant Group × Trajectory, *F*(1182.9) = 5.62, *p* = 0.019, and Group × ΔTrajectory interactions, *F*(1182.9) = 5.68, *p* = 0.018, suggesting that groups differed in trajectories before and after the knot (Figure 1). Of note, in Figure 1 the DOSE group exceeds the MCID over the inpatient stay (1).

Using the parameter estimates from Table 2, we can more precisely see these trajectories. The Usual Care group had an intercept of 24.14, with an initial trajectory (rate of change) of 19.06 points on the MoCA per year. After the knot, this rate of change substantially slowed down to 1.59 points/year. In contrast, DOSE had a similar intercept of 22.88 points, but a much greater rate of change of 65.38 points/year during the intervention. After this substantially faster rate of improvement during the intervention, DOSE had minimal change following the knot, slowing to −0.06 points/year. We modeled these effects in years to reduce scaling issues in the statistical models; however, thinking about these changes in months is more clinically practical. As shown in Figure 1, on average, Usual Care improved at a rate of about +1.59 MoCA points/month during the trial, which slowed to about +0.13 points/month following the trial. In contrast, DOSE improved at a rate of about +5.44 points/month during the intervention, which slowed to about −0.005 points/month following the intervention.

**Table 2:** Model estimates.

**Random effects**
**Outcome**	$\sigma_{\mathrm{int}}$	$\sigma_{slope}$	$\sigma_{residual}$
MoCA	3.32	*na*	2.12
**Fixed effects**			
**Parameter**	**Estimate**	**95%CI**	***p* value**
Intercept	24.14	[22.53, 25.82]	<0.001
Sex (Female)	−1.85	[−3.64, −0.07]	0.039
Age	0.01	[−0.06, 0.08]	0.809
TSS	−0.12	[−0.20, −0.03,]	0.004
Education	0.60	[0.34, 0.87]	<0.001
Trajectory	19.06	[−10.62, 49.24]	0.242
Group (Dose)	−1.26	[−3.29, 0.86]	0.226
ΔTrajectory	−17.47	[−48.49, 13.11]	0.298
Group × Trajectory	46.32	[7.50, 83.36]	0.019
Group × ΔTrajectory	−47.96	[−86.55, −8.06]	0.018

Sex centered as females +0.5/males −0.5. Other covariates were mean centered. Trajectory was centered on the first timepoint for each participant and measured in over the year. Group was coded with DOSE = 1/Usual Care = 0. MoCA, Montreal Cognitive Assessment; TSS, time from stroke to study enrollment. Group × Trajectory, trajectories before the knot (mid-intervention). Group × ΔTrajectory, trajectories changes after the knot.

DSST and Trails B similarly improved over time but were not significantly different between groups (Supplementary material).

## Discussion

The DOSE protocol was associated with increased MoCA scores during inpatient rehabilitation compared to typical rehabilitation (Figure 1). Importantly, the mixed-effect regression model has advantages over a “traditional” factorial ANOVA for these longitudinal data, including the ability to model for each participant a unique trajectory (7). As the MoCA MCID ranges from 1.22 to 2.15, our spline model showed a substantial, clinically meaningful difference, with a + 5.44 points/month improvement during the intervention compared to a + 1.59 points/month improvement with Usual Care. The placement of the knot at approximately the midpoint of the 4-week intervention may reflect the early improvements from the interdisciplinary rehabilitation practices and environment. However, there are several factors that may contribute to the knot placement. First, there is some variability in the timing of the assessments, so that not all participants were assessed at precisely 4 weeks after their baseline visit. Second, the spline provides the line of best fit, but is not required to bend at a particular data point. Notably, however, both groups plateaued substantially following the intervention (1).

There are several limitations worth considering. As this is a secondary analysis, lesion characteristics (e.g., ischemic vs. hemorrhagic, cortical vs. subcortical) were not controlled within each group, some participants had normal cognition with MoCA scores ≥26, and this is a small sample size. Thus, results should be confirmed with a larger sample where global cognition is compared with more specific aspects of cognition.

What is interesting, is that the DOSE protocol (focusing on steps and aerobic minutes) improved aspects of global cognitive function during the intervention, *without specifically targeting cognition*. Taking advantage of this early difference may lend support to continued efforts to increase intensity, during and after discharge from inpatient rehabilitation, to improve cognition.

## Data availability statement

The original contributions presented in the study are included in the article/Supplementary material, further inquiries can be directed to the corresponding author.

## Ethics statement

The studies involving human participants were reviewed and approved by University of British Columbia Research Ethics Board. The patients/participants provided their written informed consent to participate in this study.

## Author contributions

SuP, KL, TK, TL-A, SD, MB, MH, SeP, JY, and JE contributed to conception and design of the study. SuP, KL, and TK organized the data. KL performed the statistical analysis. SuP and KL wrote the first draft of the manuscript. All authors contributed to the article and approved the submitted version.

## Funding

This work was supported by Canadian Institutes of Health Research (Doctoral award TK; Operating Grant FDN143340 JE); Canada Research Chair Program (JE); Heart and Stroke Foundation Canadian Partnership for Stroke Recovery Operating Grant (JE); and Canadian Stroke Network infrastructure (MH).

## Conflict of interest

The authors declare that the research was conducted in the absence of any commercial or financial relationships that could be construed as a potential conflict of interest.

## Publisher’s note

All claims expressed in this article are solely those of the authors and do not necessarily represent those of their affiliated organizations, or those of the publisher, the editors and the reviewers. Any product that may be evaluated in this article, or claim that may be made by its manufacturer, is not guaranteed or endorsed by the publisher.

## Supplementary material

The Supplementary material for this article can be found online at: https://www.frontiersin.org/articles/10.3389/fneur.2023.1023488/full#supplementary-material

## References

[1] Wu CY Hung SJ Lin KC Chen KH Chen P Tsay PK. Responsiveness, minimal clinically important difference, and validity of the Moca in stroke rehabilitation. Occup Ther Int. (2019) 2019:2517658. doi: 10.1155/2019/2517658 31097928 PMC6487084

[2] Winstein CJ Stein J Arena R Bates B Cherney LR Cramer SC. Guidelines for adult stroke rehabilitation and recovery: a guideline for healthcare professionals from the american heart association/american stroke association. Stroke. (2016) 47:e98–e169. doi: 10.1161/STR.0000000000000098, PMID: 27145936

[3] Erickson KI Voss MW Prakash RS Basak C Szabo A Chaddock L. Exercise training increases size of hippocampus and improves memory. Proc Natl Acad Sci U S A. (2011) 108:3017–22. doi: 10.1073/pnas.1015950108, PMID: 21282661 PMC3041121

[4] Colcombe SJ Erickson KI Scalf PE Kim JS Prakash R McAuley E. Aerobic exercise training increases brain volume in aging humans. J Gerontol A Biol Sci Med Sci. (2006) 61:1166–70. doi: 10.1093/gerona/61.11.1166 17167157

[5] Klassen TD Dukelow SP Bayley MT Benavente O Hill MD Krassioukov A. Higher doses improve walking recovery during stroke inpatient rehabilitation. Stroke. (2020) 51:2639–48. doi: 10.1161/STROKEAHA.120.029245, PMID: 32811378

[6] Chiti G Pantoni L. Use of Montreal cognitive assessment in patients with stroke. Stroke. (2014) 45:3135–40. doi: 10.1161/STROKEAHA.114.004590 25116881

[7] Lohse KR Shen J Kozlowski AJ. Modeling longitudinal outcomes: a contrast of two methods. J Mot Learn Dev. (2020) 8:145–65. doi: 10.1123/jmld.2019-0007

[8] Long J. Longitudinal data analysis for the behavioral sciences using r. Thousand Oaks, CA: Sage (2016).

[9] Kuznetsova A Brockhoff PB Christensen RH. lmerTest package: tests in linear mixed effects models. J Stat Softw. (2017) 82:1–26. doi: 10.18637/jss.v082.i13

[10] Bates D Martin M Ben B Steve W. Fitting linear mixed-effects models using lme4. J Stat Softw. (2015) 67:1–48. doi: 10.18637/jss.v067.i01

