# Supplementary material for: Higher intensity walking improves global cognition during inpatient rehabilitation: a secondary analysis of a randomized control trial
Source: Front Neurol. 2023 Jun 9;14:1023488. doi: 10.3389/fneur.2023.1023488 (PMC10289188; doi:10.3389/fneur.2023.1023488)
Supplement: Supplementary file 1 [file Data_Sheet_1.pdf]

Supplement Appendix i

As discussed in the main text, we wanted to preserve variability in the time from study onset as shown in Supplemental Figure 1. Repeated Measures ANOVA (or similar) would treat the time points categorically. While this is reasonable to do conceptually (i.e., the terminal assessment is qualitatively different from a follow-up assessment), it does force a transformation onto the time variable, artificially homogenizing the data (compare the x-axis of 1A to 1B). To preserve this variability in the time variable and to more flexibly account for missing data, we chose to analyze these data using mixed-effects regression. Visual inspection of the data, as shown in 1B, revealed potential non-linearities in the data, particularly for the high dose group. As detailed below, we tested this nonlinearity using a series of polynomial and spline models.

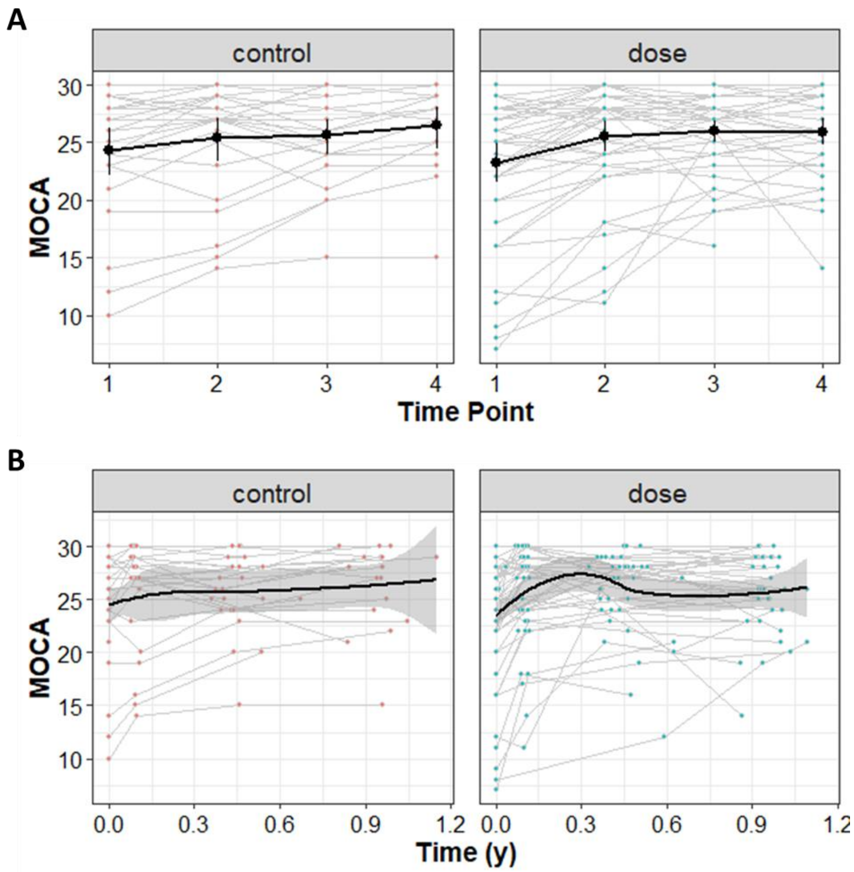

***Supplemental Figure 1**. Conceptual model of factorial ANOVA with a repeated measure of Time (Panel A) and longitudinal model with Time treated continuously (Panel B). In both panels, individual participants' data are shown with grey lines and colored dots. In Panel A, means and 95% confidence intervals at each time point are shown in black. In Panel B, a smoothed regression line is shown in black for each group, with the 95% confidence region shaded. Most critically, note that the continuous model preserves important variability in time: (1) data points are unique for all participants and (2) the first two data points are much closer together (reflecting the true length of the intervention).*

Statistical Analysis

A series of unconditional mixed-effect regression models were fit for each dependent variable to determine the best way to capture change over time. We started with a random-intercepts model, with a random-effect of participant, and then added polynomial fixed- and random-effects of time. Time was measured in years, with the 0-point set to the time of admission for each participant. Thus, the variable year.0 (used below) refers to the time since enrollment, in years, for each participant. For polynomial models, we tested linear, quadratic, and cubic fixed-effects. With the exception of Trails B models, there was a strong correlation (nearing/at 1.0) between random-intercepts and random-slopes, creating convergence issues and suggesting that the random slope parameter could be dropped from the model. Thus, for the MoCA and the DSST models included only random-intercepts, but for Trails B, models included random-intercepts and a random linear effect of year.0.

Visual inspection of the data suggested strong nonlinearities in cognitive outcomes over time. Specifically, there appeared to be a greater rate of change during the intervention, which tended to plateau following the intervention (as shown in Figure 1). To address this nonlinearity, we also fit single-knot spline models which can provide a better fit to the data than polynomial models when plateaus are present (Long, 2012). To determine the optimal knot placement, we tried two different approaches: a categorical approach and an empirical approach. For the categorical approach, we placed the knot of the spline at the terminal time-point for each participant. In this approach, all participants have the knot at the same categorical point (i.e., at the end of the intervention), but in units of year.0 that may be 0.07 y for one participant and 0.05 y for another. For the empirical approach, we recursively fit spline models with the knot in different locations using 0.01-year intervals (omitting the first and last possible times). Using Akaike's Information Criterion-corrected (AICc; Mazerolle, 2020) to assess model fit, we took the spline model with the lowest AICc as the best empirical model for each dependent variable. In this approach, all participants have a knot at precisely the same point in time (i.e., the optimal value of year.0), but individual slopes and intercepts can still vary based on the random-effects.

For each dependent variable, we thus had an intercepts-only model; linear, quadratic, and cubic polynomial models; and two different single knot linear spline models (with knots at the end of the intervention or an empirical best-fitting location). Across all dependent variables, the empirical single knot spline model provided the best model fit by AICc, with the best fitting knot consistently being placed at 0.04 years (14.6 days) across models. As shown in Supplemental Figure 2.

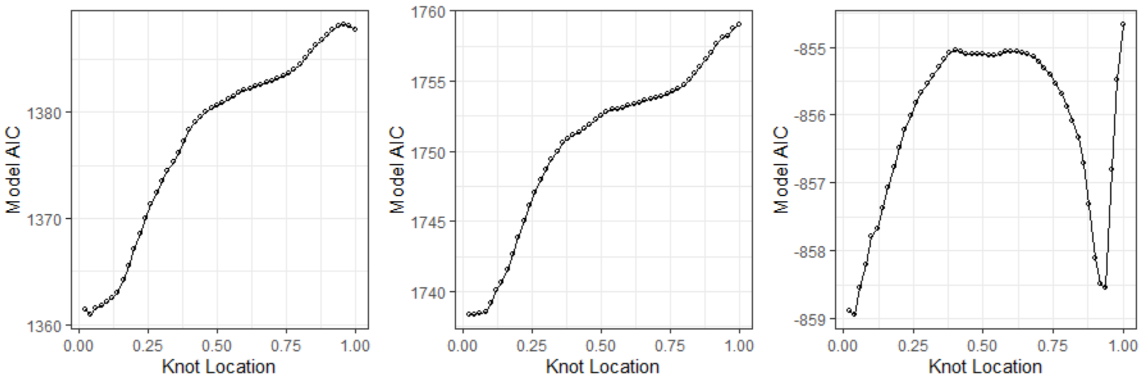

Supplemental Figure 2. *Empirical not locations for the MoCA (left), DSST (center), and Trails B (right), based on iteratively fitting spline models using different knot locations. AIC = Akaike's Information Criterion, with lower number indicating better model fit.*

Next, to test the effects of dosing on cognitive outcomes, we added fixed-effects of Group (control versus the combined high-dose groups), Group x Year.0 (group trajectories before the knot) and Group x Spline (group trajectories after the knot) interactions to the model. All models also controlled for the fixed-effects of participants' sex, age, and time since stroke as covariates. Statistical significance of the regression coefficients was based on the change in the deviance, using the Welch-Satterthwiate approximation to the degrees of freedom (Kuznetsova, Brockhoff, & Christensen, 2017), with α=0.05 for all tests. To ensure robustness of the results to violations of the model assumptions, we calculated semiparametric bootstrapped 95% confidence intervals (using n=1,000 simulations; Bates, Maechler, Bolker, & Walker, 2015).

Results

Montreal Cognitive Assessment (MoCA)

```
      Type III Analysis of Variance Table with Satterthwaite's method
                      Sum Sq Mean Sq NumDF   DenDF F value    Pr(>F)
female.c              19.769  19.769     1  71.172  4.4192  0.039075 *
age.c                  0.262   0.262     1  70.655  0.0586  0.809407
time.c                39.311  39.311     1  70.515  8.7876  0.004135 **
edu.c                 89.592  89.592     1  70.972 20.0274 2.843e-05 ***
year.0                83.575  83.575     1 182.923 18.6822 2.532e-05 ***
dose_group             6.647   6.647     1 105.799  1.4858  0.225587
spline004             75.912  75.912     1 182.943 16.9692 5.748e-05 ***
year.0:dose_group     25.149  25.149     1 182.903  5.6219  0.018778 *
dose_group:spline004 25.416  25.416     1 182.912  5.6815  0.018168 *
---
Signif. codes:  0 '***' 0.001 '**' 0.01 '*' 0.05 '.' 0.1 ' ' 1


     AIC      BIC   logLik deviance df.resid
  1270.8   1313.1   -623.4   1246.8      239

Scaled residuals:
    Min      1Q  Median      3Q     Max
-3.5681 -0.4529  0.0749  0.4785  4.2703

Random effects:
 Groups   Name        Variance Std.Dev.
 ID       (Intercept) 11.003   3.317
 Residual              4.473   2.115
Number of obs: 251, groups:  ID, 70

Fixed effects:
                            Estimate Std. Error         df t value Pr(>|t|)
(Intercept)                24.141507   0.870802 104.676277  27.723  < 2e-16
female.c                   -1.852999   0.881457  71.172054  -2.102  0.03907
age.c                       0.009084   0.037523  70.654672   0.242  0.80941
time.c                     -0.121920   0.041128  70.515126  -2.964  0.00413
edu.c                       0.601257   0.134353  70.971568   4.475 2.84e-05
year.0                     19.060405  16.239739 182.566485   1.174  0.24205
dose_groupDose             -1.263265   1.036383 105.798694  -1.219  0.22559
spline004                 -17.467057  16.735022 182.602273  -1.044  0.29798
year.0:dose_groupDose      46.323922  19.537345 182.902585   2.371  0.01878
dose_groupDose:spline004 -47.975726  20.127432 182.911907  -2.384  0.01817
---
```

Digit Symbol Substitution Test (DSST)

```
Type III Analysis of Variance Table with Satterthwaite's method
                     Sum Sq Mean Sq NumDF   DenDF F value    Pr(>F)    
female.c               0.02    0.02     1  71.358  0.0011   0.97346    
age.c                 58.86   58.86     1  71.127  3.2413   0.07604 .  
time.c               104.23  104.23     1  72.168  5.7397   0.01918 *  
edu.c                500.08  500.08     1  71.287 27.5374 1.517e-06 ***
year.0               399.91  399.91     1 180.403 22.0217 5.313e-06 ***
dose_group             4.03    4.03     1 101.887  0.2218   0.63865    
spline004            354.11  354.11     1 180.417 19.4993 1.730e-05 ***
year.0:dose_group      0.27    0.27     1 180.343  0.0150   0.90266    
dose_group:spline004   0.24    0.24     1 180.346  0.0131   0.90917    
---
Signif. codes:  0 ‘***’ 0.001 ‘**’ 0.01 ‘*’ 0.05 ‘.’ 0.1 ‘ ’ 1


     AIC      BIC   logLik deviance df.resid 
  1613.9   1656.1   -795.0   1589.9      236 

Scaled residuals: 
    Min      1Q  Median      3Q     Max 
-2.5880 -0.4075  0.0663  0.4903  5.5083 

Random effects:
 Groups   Name        Variance Std.Dev.
 ID       (Intercept) 52.24    7.228   
 Residual             18.16    4.261   
Number of obs: 248, groups:  ID, 70

Fixed effects:
                          Estimate Std. Error        df t value Pr(>|t|)
(Intercept)               19.62677    1.85951 100.02545  10.555  < 2e-16
female.c                   0.06367    1.90734  71.35762   0.033  0.97346
age.c                     -0.14635    0.08129  71.12674  -1.800  0.07604
time.c                    -0.21450    0.08953  72.16846  -2.396  0.01918
edu.c                      1.52610    0.29082  71.28672   5.248 1.52e-06
year.0                    95.28406   32.72439 179.81991   2.912  0.00405
dose_groupDose             1.04477    2.21822 101.88698   0.471  0.63865
spline004                -92.32615   33.72265 179.85066  -2.738  0.00681
year.0:dose_groupDose     -4.84756   39.57964 180.34265  -0.122  0.90266
dose_groupDose:spline004   4.65738   40.76567 180.34621   0.114  0.90917
```

Trail Making Test Part B (Trails B)

```
Type III Analysis of Variance Table with Satterthwaite's method
                        Sum Sq   Mean Sq NumDF   DenDF F value    Pr(>F)
female.c             0.0003890 0.0003890     1  70.497  0.4070 0.5255425
age.c                0.0027996 0.0027996     1  70.550  2.9294 0.0913708 .
time.c               0.0048505 0.0048505     1  69.425  5.0756 0.0274282 *
edu.c                0.0158956 0.0158956     1  74.246 16.6330 0.0001128 ***
year.0               0.0050648 0.0050648     1 127.728  5.2998 0.0229467 *
dose_group           0.0000051 0.0000051     1  88.858  0.0053 0.9422025
spline004            0.0041048 0.0041048     1 128.072  4.2952 0.0402261 *
year.0:dose_group    0.0003894 0.0003894     1 127.713  0.4074 0.5244267
dose_group:spline004 0.0004198 0.0004198     1 128.056  0.4393 0.5086582
---

     AIC      BIC   logLik deviance df.resid
  -804.9   -755.8    416.4   -832.9      232

Scaled residuals:
    Min      1Q  Median      3Q     Max
-1.7760 -0.5222 -0.0187  0.4435  4.7619

Random effects:
 Groups   Name        Variance  Std.Dev. Corr
 ID       (Intercept) 0.0025125 0.05012
          year.0      0.0006784 0.02605  -0.02
 Residual             0.0009557 0.03091
Number of obs: 246, groups:  ID, 70

Fixed effects:
                           Estimate Std. Error         df t value Pr(>|t|)
(Intercept)               1.058e-01  1.319e-02  8.813e+01   8.017 4.24e-12
***
female.c                 -8.560e-03  1.342e-02  7.050e+01  -0.638 0.525542
age.c                    -9.793e-04  5.722e-04  7.055e+01  -1.712 0.091371
time.c                   -1.407e-03  6.247e-04  6.942e+01  -2.253 0.027428
edu.c                     8.480e-03  2.079e-03  7.425e+01   4.078 0.000113
year.0                    4.321e-01  2.446e-01  1.278e+02   1.767 0.079613
dose_groupDose            1.139e-03  1.567e-02  8.886e+01   0.073 0.942202
spline004                -4.142e-01  2.521e-01  1.281e+02  -1.643 0.102886
year.0:dose_groupDose    -1.876e-01  2.939e-01  1.277e+02  -0.638 0.524427
dose_groupDose:spline004  2.008e-01  3.029e-01  1.281e+02   0.663 0.508658
```

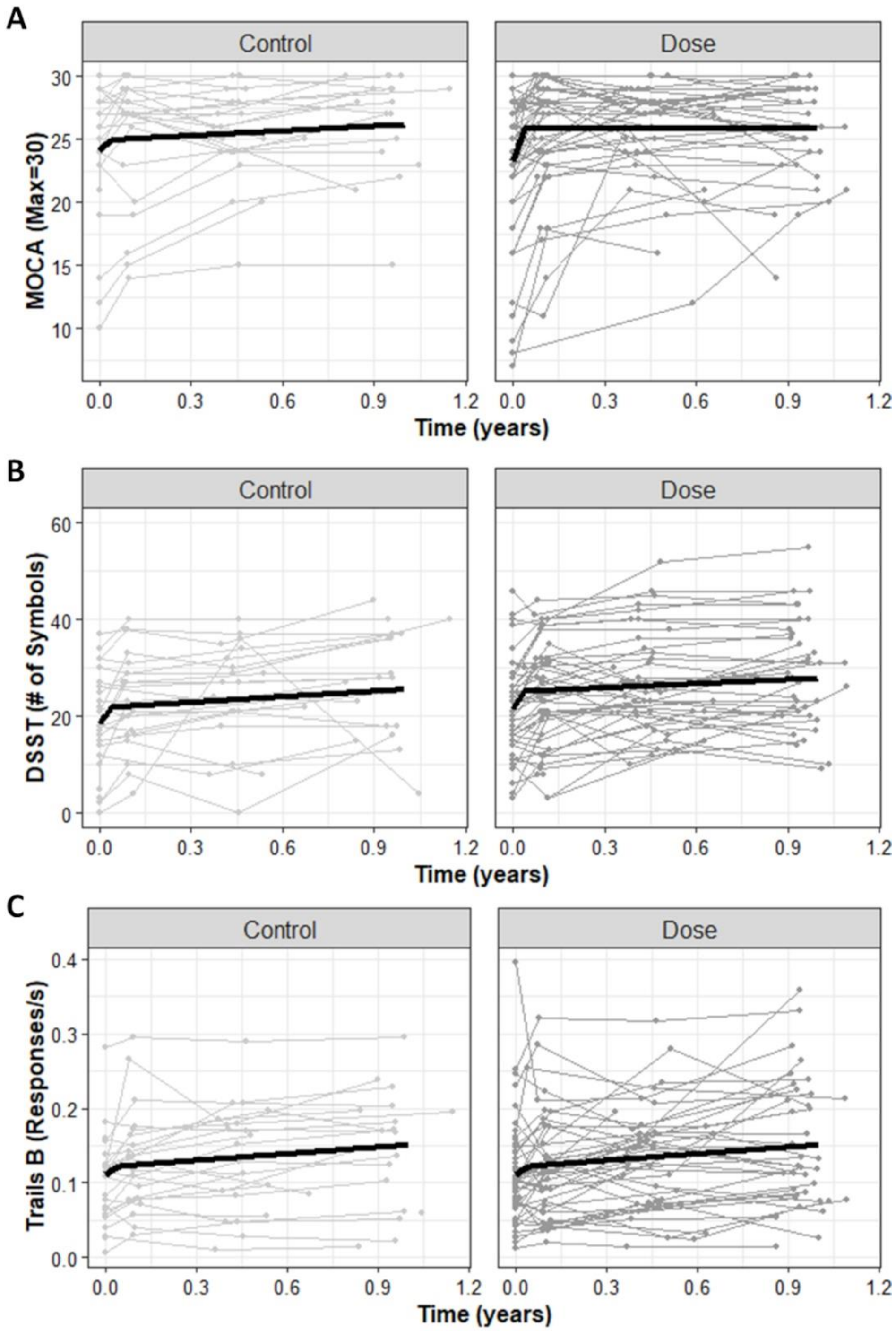

***Supplemental Figure 3**. Individual trajectories (grey) and marginal predictions from the fixed-effects spline model (black) for the MoCA (A), DSST (B), and the Trails-B (C).*
